# Long-term symptoms in children after a *Cryptosporidium hominis* outbreak in Sweden: a 10-year follow-up

**DOI:** 10.1007/s00436-025-08455-7

**Published:** 2025-01-25

**Authors:** Marije Boks, Mikael Lilja, Anna Lindam, Micael Widerström, Angelica Persson, Pontus Karling, Malin Sjöström

**Affiliations:** 1 Department of Public Health and Clinical Medicine, Umeå University, Umeå, Sweden; 2 Department of Public Health and Clinical Medicine, Unit of Research, Education and Development–Östersund, Umeå University, Umeå, Sweden; 3 Department of Clinical Microbiology, Umeå University, Umeå, Sweden

**Keywords:** *Cryptosporidium*, Disease outbreaks, Sequelae, Post-infectious symptoms, Paediatric infections

## Abstract

In 2010, a *Cryptosporidium* *hominis* outbreak resulted in 27,000 clinical cryptosporidiosis cases (45% of the population) in Östersund, Sweden. Long-term abdominal and joint symptoms are common following cryptosporidiosis in adults, and it can affect the development of children in low-income countries. We investigated the potential consequences for children in a high-income setting. In 2011, we prospectively surveyed 600 randomly selected children aged 0–5 years from Östersund. Cases were defined as respondents reporting new episodes of diarrhoea during the outbreak. After 10 years, respondents received a follow-up questionnaire about long-term symptoms (*n* = 423). We used *X*^2^ and Mann–Whitney *U* tests to assess between-group differences in demographics and the mean number of symptoms. Logistic regressions adjusted for sex, age, and prior issues with loose stools were used to examine associations between case status and symptoms reported at follow-up. We retrieved data on healthcare visits from patient records. In total, 121 cases and 174 non-cases responded to the follow-up questionnaire (69.7%). Cases reported 1.74 (median 1.00, range 0–14) symptoms and non-cases 1.37 (median 0.00, range 0–11) symptoms (*p* = 0.029). Cases were more likely to report joint symptoms (aOR 4.0, CI 1.3–12.0) and fatigue (aOR 1.9, CI 1.1–3.4), but numbers were generally low. We found no between-group differences in abdominal symptoms, healthcare utilization, or disease diagnoses. Children aged 0–5 years from high-income countries may experience long-term symptoms after cryptosporidiosis, but may not be affected to the same extent as adults or their peers living in low-income countries.

**Supplementary Information:**

The online version contains supplementary material available at 10.1007/s00436-025-08455-7.

## Background

The parasite *Cryptosporidium* is a significant cause of diarrhoea worldwide (Carter 2020, Bourli 2023). A vast majority of human cases are caused by either *Cryptosporidium hominis* or *Cryptosporidium parvum*, and infection typically occurs through the consumption of contaminated water or food (Bouzid 2013; Checkley^ 2015^; Feng 2018). Young children and immunocompromised individuals are especially vulnerable to severe infection (Chalmers 2013).

Few large *Cryptosporidium* outbreaks have been described in the literature. The largest outbreak hit Milwaukee, WI (USA), in 1993, affecting more than 400,000 individuals (Mac Kenzie 1994). In November 2010, the inhabitants of Östersund, Sweden, faced a large outbreak of *C. hominis* subtype IbA10G2 due to contaminated drinking water (Widerström 2014). Approximately 27,000 individuals, 45% of the city’s population, contracted clinical cryptosporidiosis. Details on the outbreak and related investigations have previously been published elsewhere (Widerström 2014).

In adults, cryptosporidiosis has been associated with long-term symptoms up to 10 years after the initial infection, especially abdominal and joint symptoms (Boks 2023). In addition, gastrointestinal infections in general have been associated with irritable bowel syndrome (IBS), inflammatory bowel disease (IBD), and other autoimmune disorders (Rogler 2016; Axelrad 2020; Pogreba-Brown 2020). Following the outbreak in Östersund, the incidence of IBD in the area increased among individuals aged 40 years and older (incidence rate ratio 1.69, CI 1.13–2.5) (Boks^ 2022^).

Research on possible long-term consequences of cryptosporidiosis in children is limited. Most published studies have been conducted in low-income countries, where cryptosporidiosis has been associated with faltering growth and decreased cognitive function (Kotloff 2013; Ajjampur 2010). However, *Cryptosporidium* is also a common cause of childhood diarrhoea in high-income countries (Skovgaards 2018). Surprisingly, there are no published reports on the long-term effects on children in these regions. Although children have been included in some follow-up studies, they were not analysed separately (Sjöström 2022; Carter 2019; Igloi 2018; Insulander 2013; Hunter 2004).

We hypothesized that young children from Östersund who suffered from clinical cryptosporidiosis during the outbreak would experience long-term abdominal and joint symptoms 10 years later, in line with previous findings in adults. We also hypothesized that they would have a higher healthcare utilization as well as a higher prevalence of auto-immune disorders.

## Methods

We performed a prospective cohort study based on a 10-year follow-up of children from Östersund, Sweden, who were aged 0–5 years during the *C. hominis* outbreak from 1 November 2010 through 31 January 2011.

### Study population and data collection

#### ***Questionnaire***

In late January 2011, 600 randomly selected children aged 0–5 years (born 2005 or later) residing in Östersund municipality received a questionnaire (hereafter, outbreak questionnaire) by post. Parents/custodians were asked to complete the questionnaire for the children. It included items regarding demographics, cryptosporidiosis symptoms, and medical conditions. In addition, the parents were asked if their child had experienced long-term or recurring issues with loose stools, abdominal pain, and/or bloating during the 2 years prior to the outbreak. The questionnaire was returned for 426 children (71.0%).

**Fig. 1:**
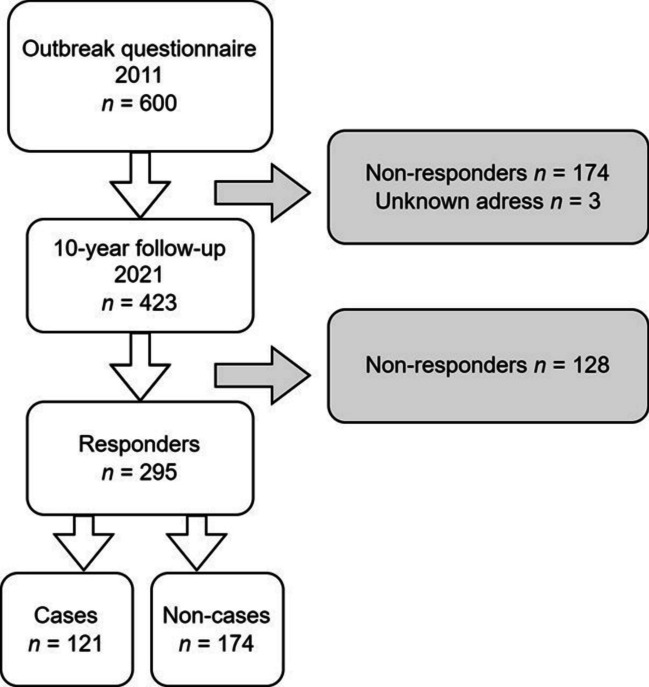
Case selection. Case: new episodes of diarrhoea (≥ 3 loose stools a day and/or watery diarrhoea) between 1 November 2010 and 31 January 2011, in respondent living in Östersund municipality mid-January 2011. Non-case: respondent not fulfilling the case criteria

In January 2021, a follow-up questionnaire (Supplement 1) was sent by post to the respondents to the outbreak questionnaire for whom the current address was known (*n* = 423, Fig. 1). The mailing included a pre-paid envelope to return the questionnaire, and a reminder was sent after 1 month. The parents/custodians were asked to report whether the child had experienced the following possible post-infectious symptoms during the last 3 months: loose stools, watery diarrhoea, bloody diarrhoea, change in bowel habits, abdominal pain, bloating, nausea, vomiting, heartburn, loss of appetite, weight loss, headache, eye pain, fatigue, any stiff joints, joint pain, swollen joints, and/or joint discomfort. They were also asked to state the presence of IBS, IBD, gluten intolerance, lactose intolerance, cow’s milk allergy, other food allergies, diabetes, and rheumatic disease, as well as the use of antacids and systemic corticosteroids. The returned questionnaires were optically scanned and then transformed into a data file.

#### *Case definition*

Case definition was based on self-reported symptoms. A case was defined as a respondent who, on the outbreak questionnaire, reported new episodes of diarrhoea (≥ 3 loose stools daily) and/or watery diarrhoea with onset between 1 November 2010 and 31 January 2011 (World Health Organization 2017). Respondents of the outbreak questionnaire who did not fulfil these criteria were defined as non-cases and were unmatched controls to the cases.

#### *Composite outcomes*

We created composite outcomes when reported numbers were low or where there was considerable overlap in symptoms reported:

- *previous abdominal issues* included all respondents reporting loose stools, abdominal pain, or bloating prior to the outbreak;
- *previous food allergy or intolerance* included all respondents reporting cow’s milk allergy, lactose intolerance, gluten intolerance, or other food-related allergy prior to the outbreak;
- *diarrhoea* included loose stools, watery diarrhoea, and bloody diarrhoea as reported during follow-up;
- *joint symptoms* included joint discomfort, stiff joints, joint pain, and swollen joints as reported during follow-up.

#### *Patient records*

We collected data on outpatient visits to primary care (not including routine visits to the children’s health centre) and the departments of paediatrics and child psychiatry to investigate both somatic and psychological well-being. The data was retrieved from the healthcare region Jämtland Härjedalen for the period 1 January 2011–31 December 2020, for all respondents to the follow-up questionnaire. The collected data included International Classification of Diseases (ICD) codes, visit date and time, type of visit (in-person or virtual consultation), and care provider category (doctor; nurse; licensed psychological staff, including counsellor, psychologist, psychotherapist; team visit, which was any combination of healthcare providers with visits registered at the same date and time).

We also looked into predefined “chronic” conditions, for which receiving the diagnosis once throughout the study period sufficed. Chronic conditions included type 1 diabetes (ICD code E10), lactose intolerance (E73), pollen allergy (J30), asthma (J45), Crohn’s disease (K50), ulcerative colitis (K51), celiac disease (K90.0, K900), and chronic juvenile arthritis (M08). In addition, we noted recurring conditions common in childhood, for which we counted the number of diagnoses per year. Recurring conditions included gastrointestinal infections (A0), upper airway infections (J0), otitis media (H65, H66), IBS (K58), joint pain (M25), and abdominal pain (R10).

### Statistical analyses

Outcome measures were analysed by case status, sex, and age at the time of the outbreak (0–2 or 3–5 years, as previous studies have shown that the youngest children are most vulnerable). *X*^2^ tests and Mann–Whitney *U* tests were used to assess between-group differences in demographic variables and the mean number of symptoms. Logistic regression analyses were used to examine associations between case status and symptoms reported on the follow-up questionnaire. The results were adjusted for age (category), sex, and previous history of loose stools, and then presented as odds ratios (ORs) with 95% confidence intervals (CIs).

Healthcare visits for cases and non-cases were compared using Mann–Whitney *U* tests. Possible between-group differences in diagnoses were compared using *X*^2^ tests and Mann–Whitney *U* tests.

All statistical calculations were performed using IBM SPSS Statistics for Windows, version 29 (IBM Corp., Armonk, NY, USA). Missing values were excluded from analyses. The significance level was set at 0.05.

## Results

A total of 295 children (69.7%) responded to the 10-year follow-up questionnaire (Fig. 1). A dropout analysis showed that age and sex did not differ between responders and non-responders. However, non-responders were more often defined as cases (71/128 or 55.5%) compared to responders (121/295 or 41.0%) (*p* = 0.006).

A total of 121 respondents were defined as cases and 174 as non-cases (Table 1). There were no significant differences in sex or age between the groups, but cases more often reported on the outbreak questionnaire long-term or recurrent issues with loose stools during the 2 years prior to the outbreak. No differences were observed in the prevalence of self-reported abdominal pain, bloating, cow’s milk allergy, lactose intolerance, gluten intolerance, or other food intolerances pre-outbreak. Cases and non-cases did not differ in their self-reported use of medications or other comorbidities at baseline or follow-up.

**Table 1:** Demographic characteristics of the study population

Characteristic	Cases *n* (%)	Non-cases *n* (%)	Total *n* (%)	*p*-value
Total cases	121	174	295	
Sex				0.284
Male	64 (52.9)	81 (46.6)	145 (49.2)	
Female	57 (47.1)	93 (53.4)	150 (50.8)	
Age at outbreak, years				0.057
0–2	72 (59.5)	84 (48.3)	156 (52.9)	
3–5	49 (40.5)	90 (51.7)	139 (47.1)	
Previous abdominal issues^a^	23 (19.0)	13 (7.5)	36 (12.2)	0.003*
Loose stools	16 (13.2)	11 (6.3)	27 (9.2)	0.043*
Abdominal pain	9 (7.4)	8 (4.6)	17 (5.8)	0.303
Bloating	4 (3.3)	7 (4.0)	11 (3.7)	0.749
Previous food allergy or intolerance^b^	9 (7.4)	9 (5.2)	18 (6.1)	0.439

^a^Respondents reporting lasting or recurrent episodes of loose stools, abdominal pain, and/or bloating in the 2 years prior to the outbreak

^b^Respondents reporting cow’s milk allergy, lactose intolerance, gluten intolerance, and/or other food-related allergy

*Statistically significant

### Reported symptoms after 10 years

The mean number of symptoms reported by cases was 1.74 (median 1.00, range 0–14), whereas non-cases reported 1.37 symptoms (median 0.00, range 0–11; *p* = 0.029). Logistic regressions adjusted for sex, age, and previous issues with loose stools showed increased ORs for joint symptoms (OR 4.0, CI 1.3–12.0) and fatigue (OR 1.9, CI 1.1–3.4) (Table 2). Symptoms reported by fewer than 5 individuals per group and subtypes of diarrhoea and joint symptoms are not provided because the uncertainty in the estimates was too great.

**Table 2:** Symptoms reported 10 years after the outbreak among cases and non-cases

**Outcome**		**Cases** ***n* = 121 (41.0%)**	**Non-cases** ***n* = 174 (59.0%)**	**aOR** **(95% CI)**
Diarrhoea^a^		9 (7.4)	9 (5.2)	1.4 (0.5–3.6)
Constipation		10 (8.3)	19 (10.9)	0.6 (0.3–1.4)
Changes in bowel habits		13 (10.7)	14 (8.0)	1.2 (0.5–2.8)
Abdominal pain		22 (18.2)	22 (12.6)	1.4 (0.7–2.7)
Bloating		15 (12.4)	13 (7.5)	1.7 (0.8–3.7)
Heartburn		6 (5.0)	11 (6.3)	0.8 (0.3–2.2)
Nausea		20 (16.5)	25 (14.4)	1.1 (0.6–2.2)
Joint symptoms^b^		11 (9.1)	5 (2.9)	4.0 (1.3–12.0)*
Headache		54 (44.6)	57 (32.8)	1.6 (1.0–2.7)
Ocular pain		9 (7.4)	13 (7.5)	1.1 (0.4–2.6)
Fatigue		32 (26.4)	27 (15.5)	1.9 (1.1–3.4)*
Fever		13 (10.7)	11 (6.3)	1.9 (0.8–4.3)
Loss of appetite		5 (4.1)	10 (5.7)	0.7 (0.2–2.2)

Logistic regressions, adjusted for sex, age, and issues with loose stools prior to the outbreak. *aOR*, adjusted odds ratio; *CI*, confidence interval

^a^Composite outcome including all individuals reporting loose stools, watery diarrhoea, and/or bloody diarrhoea

^b^Composite outcome including all individuals reporting any stiff joints, joint pain, swollen joints, and/or joint discomfort

*Statistically significant

### Healthcare utilization

**Fig. 2:**
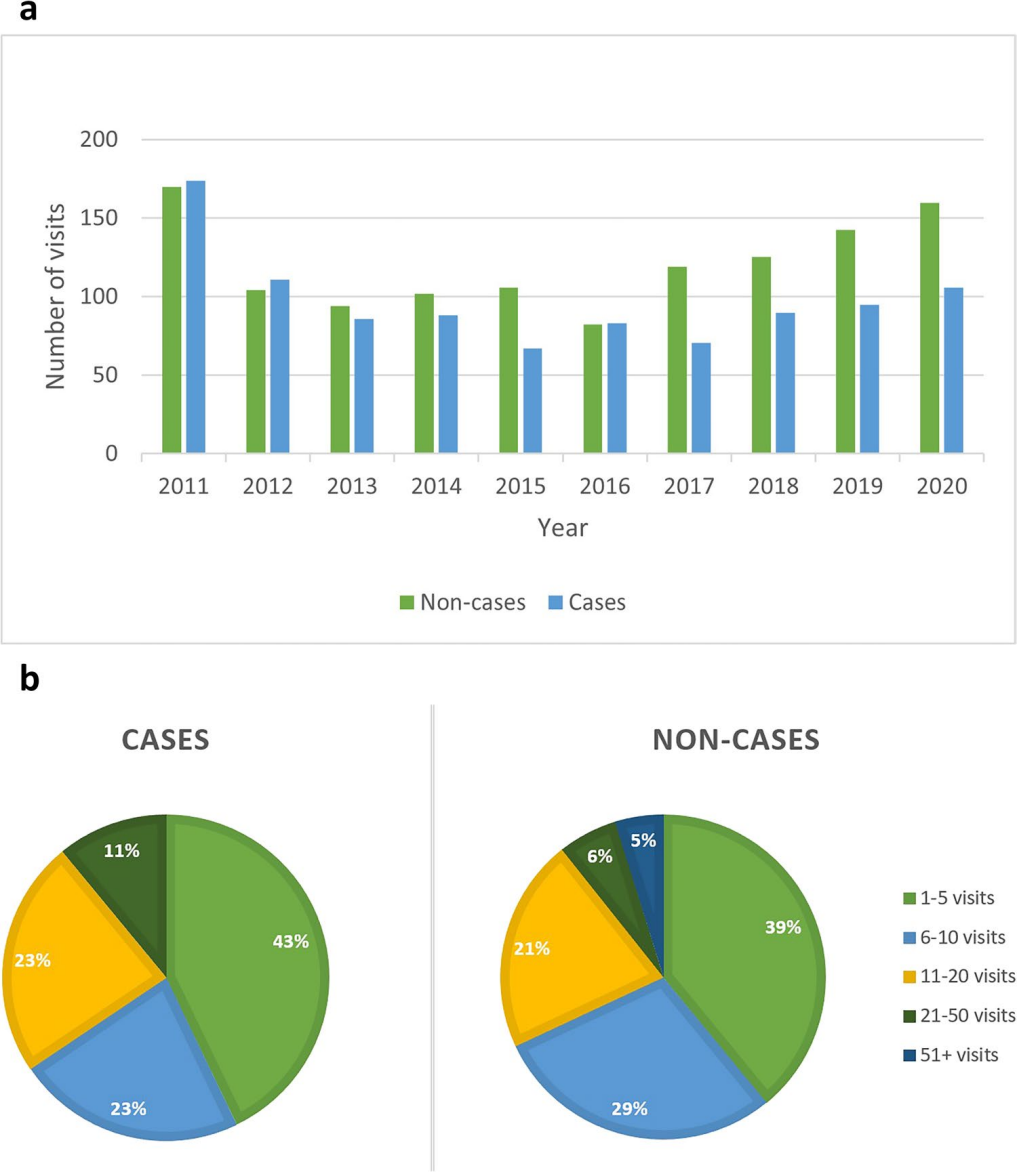
Number of healthcare visits in 2011–2020. **a** Per year by case status adjusted to per 100 participants. **b** Number of visits by case status

Data from the electronic patient records was available for 119/121 cases and 169/174 non-cases. There were no visits recorded for the remaining 7 individuals, suggesting that they either did not visit the included departments during the study period, or moved to another healthcare region before they made a first post-outbreak healthcare visit. A total of 3191 visits were recorded. For the entire follow-up period, 970 visits per 100 participants were registered for the cases (median 7.0, range 1–45) and 1205 visits per 100 participants (median 7.0, range 1–185) for non-cases (Fig. 2). Most visits were to primary care; 571 per 100 cases (median 5.00, range 0–21) and 527 per 100 non-cases (median 5.00, range 1–19). We found no differences between cases and non-cases when comparing the number of visits for the entire study period or per year, per department (Supplement 2), or per care provider category.

### Diagnoses

**Fig. 3:**
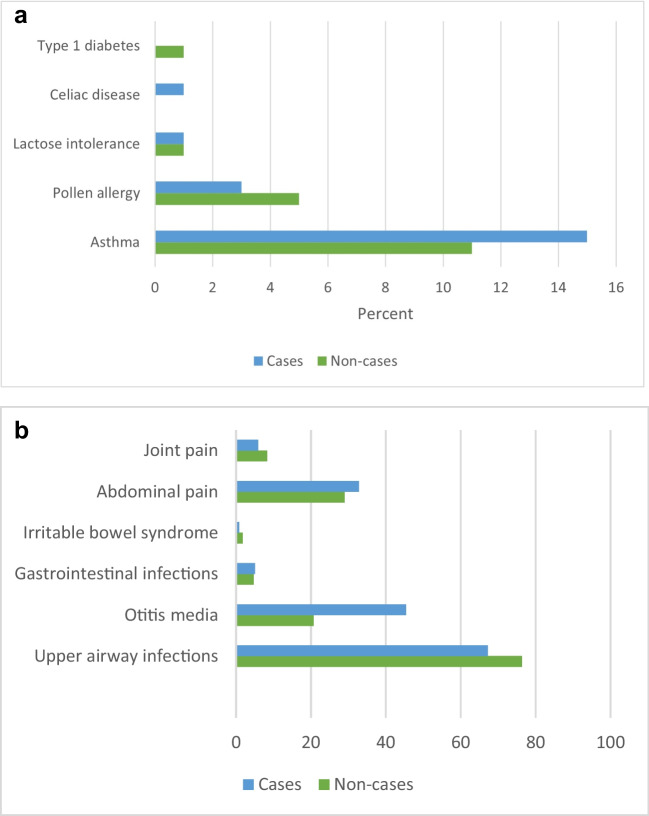
Chronic and recurring diagnoses by case status. **a** Chronic diagnoses, percentage of participants with the diagnosis. **b** Recurring diagnoses, number of diagnoses in 2011–2020 adjusted to per 100 participants

During the follow-up period, none of the participants was diagnosed with Crohn’s disease, ulcerative colitis, or chronic juvenile arthritis. The number of individuals affected by the remaining chronic conditions was generally low, and no differences were found between cases and non-cases. We also did not observe differences between cases and non-cases regarding the diagnosis of common childhood infections, abdominal pain, or joint pain (Fig. 3).

## Discussion

This prospective cohort study involved a 10-year follow-up of children aged 0–5 years during the *Cryptosporidium hominis* outbreak in Östersund in 2010. Cases reported experiencing joint symptoms and fatigue more frequently than non-cases. However, we did not observe differences between cases and non-cases in the reporting of abdominal symptoms, the prevalence of autoimmune diseases, the incidence of common childhood conditions, or the number of healthcare visits.

### Results in context

Even though cases reported more symptoms after 10 years, our results do not demonstrate the pronounced consequences for the children as previous studies have in low-income countries (Khalil 2018; Kotloff 2013; Ajjampur 2010; Guerrant 1999). Several explanations are possible. First, well-nourished and generally healthy children may be sickened less by the acute infection, as cryptosporidiosis is more prevalent in malnourished children (Madadi 2020; Bouzid 2013; Chalmers 2013). Second, in some studies, the ingestion of larger numbers of oocysts was related to more severe disease, which could be a factor for long-term symptoms (Genta 1993; Clayton 1994). It is possible that affected children in low-income countries generally ingest larger numbers of oocysts, whereas the density of oocysts in the drinking water in Östersund during the outbreak was relatively low (Widerström 2014).

Since cryptosporidiosis is associated with growth faltering in children in low-income countries, it would have been interesting to analyse height even in our cohort. Unfortunately, growth data is mainly registered by school nurses in decentralized records, which we did not have access to. However, we do not expect that a single episode of gastroenteritis does have a significant long-term effect on neither growth or cognitive functioning in otherwise healthy, well-fed children in a high-income country.

Low numbers and wide confidence intervals show that our results should be interpreted with caution. However, they are in line with a systematic review on long-term symptoms after cryptosporidiosis, as well as our findings in a 10-year follow-up of adults after the outbreak in Östersund (Carter 2020; Boks 2023). In addition to fatigue and joint symptoms, adults also reported headaches and abdominal symptoms such as diarrhoea, changed bowel habits, and abdominal pain. A prospective study from Wales found that the proportion of individuals that had developed IBS (-like) symptoms 1 year after cryptosporidiosis was higher in children than in adults, suggesting that children can also develop abdominal sequelae (Carter 2019). Chronic fatigue and IBS were also found 10 years after an outbreak of the parasite *Giardia lamblia* in Bergen, Norway, though the number of children included in this study was low (Litleskare 2018). Insufficient statistical power may explain why we did not find differences in abdominal symptoms in this study.

The pathophysiology underlying persistent post-infectious symptoms is only partially understood. Infection-induced alterations in the intestinal microbiota can lead to intestinal barrier dysfunction, visceral hypersensitivity, and immune system imbalance. These changes may also disrupt gut-brain axis communication and trigger low-grade inflammation, potentially resulting in IBS-like symptoms, fatigue, and other extraintestinal symptoms (Berumen 2021, Ohlsson 2022, Lupu 2024). As we do not exactly know which factors play a major role in the development of long-term symptoms after cryptosporidiosis, we cannot be certain that the results from this study are applicable to other high-income countries.

Our study found no significant differences between cases and non-cases when looking at diagnoses and number of healthcare visits, indicating that the long-term symptoms experienced to some extent by cases were not a cause for increased illness or healthcare utilization.

We made some considerations in the analysis of the number of visits. Outpatient visits were usually in-person, but sometimes virtual (telephone or video consultation). We decided not to analyse these types of outpatient visits separately. The choice of a virtual visit was probably influenced by department routines, the distance between the patient’s home and the hospital, and the SARS-CoV-2 pandemic in 2020, none of which is expected to be relevant for our outcomes. Short phone calls to, for example, inform about test results, are not registered as outpatient visits and, therefore, were not included in this study.

The number of visits per 100 participants varied between cases and non-cases, particularly for the department of child psychiatry. This difference was driven by a small number of outliers with an exceptionally high number of visits. Excluding these outliers from the analysis did not result in significantly different findings.

Following the *Cryptosporidium* outbreak in Östersund, an increased incidence of IBD was observed in the population aged ≥ 40 years (incidence rate ratio 1.69, CI 1.13–2.5) (Boks 2022). However, this increase was not observed in our follow-up of adults, likely due to insufficient statistical power (Boks 2023). In our child cohort, no IBD diagnoses were registered a decade after the outbreak. This finding is not unexpected, given the cohort size and the estimated prevalence of childhood-onset IBD in Sweden, which is 75 per 100,000 inhabitants (Ludvigsson 2017). Furthermore, the majority of the cohort had not yet reached the median age of childhood-onset IBD diagnosis in Sweden, which is 14 years, providing an additional explanation for the absence of IBD diagnoses (Mouratidou 2023).

### Strengths and limitations

The strengths of this study are its prospective design and randomly selected cohort. It was conducted by a qualified research team with backgrounds in general practice, gastroenterology, and infectious diseases and supported by an experienced statistician. To date, it is the largest study with the longest follow-up of children after a *C. hominis* infection in a high-income country. Response rates were high for both the outbreak and follow-up questionnaires.

Somewhat unexpectedly, non-responders were more frequently classified as cases (55.5%) compared to responders (41.0%), which may have potentially weakened our results. We do not have a reasonable explanation as to why this was the case.

The main limitation of this study is that case status was based on self-reported symptoms instead of laboratory-confirmed diagnoses. Only 149 inhabitants of the region had their infections confirmed by faecal microscopy. These individuals were primarily those who became ill early in the outbreak or required hospital care. Testing of the general population was discontinued once the pathogen responsible for the outbreak was identified. The same strain of *C. hominis* was identified in a subset of these faecal samples, as well as in water samples, and no other pathogens were detected, making it plausible that the symptoms reported by our study cohort were caused by *C. hominis* (Widerström 2014). To reduce the risk of misclassification, we decided beforehand that individuals with a diagnosis of IBD or IBS prior to the outbreak would be excluded from the analysis, but none of the respondents had such a diagnosis.

It is possible that misclassification occurred due to the presence of asymptomatic individuals. A Belgian study on a *Cryptosporidium* outbreak in a day-care centre reported that 9/38 (24%) children were asymptomatic (Vandenberg 2012). Even though there is evidence that asymptomatic cryptosporidiosis in young children from low-income countries can affect growth in the months following the infection, whether asymptomatic infection can cause sequelae is not known (Khalil 2018; Das 2023). If this is the case and similar rates apply to our population, it could have weakened our results.

We lack information on infection status during or before follow-up. Therefore, it is possible that some individuals reported symptoms during follow-up that were the result of gastrointestinal disease or infection rather than long-term symptoms resulting from cryptosporidiosis. However, this possibility applies to both cases and non-cases and is not expected to significantly influence our outcomes. During the follow-up period, no significant outbreaks with gastrointestinal pathogens were reported in the area.

We were not able to collect data from other healthcare regions and lacked data on visits outside Region Jämtland Härjedalen. Only one participant moved from the region during the study period (in 2014), and seeking routine care outside one’s own region is both uncommon and unlikely due to large distances. This is the situation for both cases and non-cases and, therefore, is not expected to affect our outcomes.

### Future research and clinical implications

Further research is needed to better understand the long-term effects of *Cryptosporidium* infection, especially in children from high-income countries. A future follow-up of this cohort could provide further insights into persisting post-infectious symptoms and the potential development of autoimmune diseases that may not have emerged during this 10-year follow-up period. Additionally, it could include an analysis of educational attainment and/or socioeconomic status as proxies for cognitive functioning. Investigating the mechanisms underlying symptom persistence could aid in developing effective treatment options. This research is especially important given the anticipated increase in *Cryptosporidium* outbreaks due to climate change (Young 2015).

## Conclusions

Clinical cryptosporidiosis in children up to 5 years of age from a high-income country was associated with increased reports of fatigue and joint symptoms 10 years later, which is in concordance with previous findings in adults. However, these children did not experience more abdominal symptoms, nor did they have higher healthcare utilization or different diagnoses than unaffected peers. Due to generally low numbers, the results should be interpreted with caution. Nonetheless, the findings suggest that children may not be affected to the same extent as adults, and that children from high-income countries may not be as affected as children from low-income countries. However, prioritizing adequate water treatment remains essential worldwide.

## Supplementary Information

Below is the link to the electronic supplementary material.

Supplementary file1 (PDF 162 KB)

Supplementary file2 (JPG 482 KB)

## Data Availability

The datasets used during the current study are available from the corresponding author upon reasonable request.

## References

[CR1] Ajjampur SS, Sarkar R, Sankaran P, Kannan A, Menon VK, Muliyil J et al (2010) Symptomatic and asymptomatic *Cryptosporidium* infections in children in a semi-urban slum community in southern India. Am J Trop Med Hyg 83:1110–1115. 10.4269/ajtmh.2010.09-0644 21036847 10.4269/ajtmh.2010.09-0644 PMC2963979

[CR2] Axelrad JE, Cadwell KH, Colombel JF, Shah SC (2020) Systematic review: gastrointestinal infection and incident inflammatory bowel disease. Aliment Pharmacol Ther 51:1222–1232. 10.1111/apt.15770 32372471 10.1111/apt.15770 PMC7354095

[CR3] Berumen A, Edwinson AL, Grover M (2021) Post-infection irritable bowel syndrome. Gastroenterol Clin North Am 50:445–461. 10.1016/j.gtc.2021.02.007 34024451 10.1016/j.gtc.2021.02.007 PMC8144546

[CR4] Boks M, Lilja M, Widerström M, Karling P, Lindam A, Eriksson A et al (2022) Increased incidence of late-onset inflammatory bowel disease and microscopic colitis after a *Cryptosporidium hominis* outbreak. Scand J Gastroenterol 57:1443–1449. 10.1080/00365521.2022.2094722 35802626 10.1080/00365521.2022.2094722

[CR5] Boks M, Lilja M, Widerström M, Karling P, Lindam A, Sjöström M (2023) Persisting symptoms after *Cryptosporidium hominis* outbreak: a 10-year follow-up from Östersund, Sweden. Parasitol Res 122:1631–1639. 10.1007/s00436-023-07866-8 37199767 10.1007/s00436-023-07866-8 PMC10193336

[CR6] Bourli P, Eslahi AV, Tzoraki O, Karanis P (2023) Waterborne transmission of protozoan parasites: a review of worldwide outbreaks - an update 2017–2022. J Water Health 21:1421–1447. 10.2166/wh.2023.094 37902200 10.2166/wh.2023.094

[CR7] Bouzid M, Hunter PR, Chalmers RM, Tyler KM (2013) *Cryptosporidium* pathogenicity and virulence. Clin Microbiol Rev 26:115–134. 10.1128/CMR.00076-12 23297262 10.1128/CMR.00076-12 PMC3553671

[CR8] Carter BL, Stiff RE, Elwin K, Hutchings HA, Mason BW, Davies AP et al (2019) Health sequelae of human cryptosporidiosis-a 12-month prospective follow-up study. Eur J Clin Microbiol Infect Dis 38:1709–1717. 10.1007/s10096-019-03603-1 31302785 10.1007/s10096-019-03603-1

[CR9] Carter BL, Chalmers RM, Davies AP (2020) Health sequelae of human cryptosporidiosis in industrialised countries: a systematic review. Parasit Vectors 13:443. 10.1186/s13071-020-04308-7 32887663 10.1186/s13071-020-04308-7 PMC7650228

[CR10] Chalmers RM, Katzer F (2013) Looking for *Cryptosporidium*: the application of advances in detection and diagnosis. Trends Parasitol 29:237–251. 10.1016/j.pt.2013.03.001 23566713 10.1016/j.pt.2013.03.001 PMC7106352

[CR11] Checkley W, White AC Jr, Jaganath D, Arrowood MJ, Chalmers RM, Chen XM et al (2015) A review of the global burden, novel diagnostics, therapeutics, and vaccine targets for *Cryptosporidium*. Lancet Infect Dis 15:85–94. 10.1016/S1473-3099(14)70772-8 25278220 10.1016/S1473-3099(14)70772-8 PMC4401121

[CR12] Clayton F, Heller T, Kotler DP (1994) Variation in the enteric distribution of cryptosporidia in acquired immunodeficiency syndrome. Am J Clin Pathol 102:420–425. 10.1093/ajcp/102.4.420 7942597 10.1093/ajcp/102.4.420

[CR13] Das R, Palit P, Haque MA, Levine MM, Kotloff KL, Nasrin D et al (2023) Symptomatic and asymptomatic enteric protozoan parasitic infection and their association with subsequent growth parameters in under five children in South Asia and sub-Saharan Africa. PLoS Negl Trop Dis 17:e0011687. 10.1371/journal.pntd.0011687 37816031 10.1371/journal.pntd.0011687 PMC10588856

[CR14] Feng Y, Ryan UM, Xiao L (2018) Genetic diversity and population structure of *Cryptosporidium*. Trends Parasitol 34:997–1011. 10.1016/j.pt.2018.07.009 30108020 10.1016/j.pt.2018.07.009

[CR15] Genta RM, Chappell CL, White AC Jr, Kimball KT, Goodgame RW (1993) Duodenal morphology and intensity of infection in AIDS-related intestinal cryptosporidiosis. Gastroenterology 105:1769–1775. 10.1016/0016-5085(93)91075-s 8253352 10.1016/0016-5085(93)91075-s

[CR16] Guerrant DI, Moore SR, Lima AA, Patrick PD, Schorling JB, Guerrant RL (1999) Association of early childhood diarrhea and cryptosporidiosis with impaired physical fitness and cognitive function four-seven years later in a poor urban community in northeast Brazil. Am J Trop Med Hyg 61:707–713. 10.4269/ajtmh.1999.61.707 10586898 10.4269/ajtmh.1999.61.707

[CR17] Hunter PR, Hughes S, Woodhouse S, Raj N, Syed Q, Chalmers RM et al (2004) Health sequelae of human cryptosporidiosis in immunocompetent patients. Clin Infect Dis 39:504–510. 10.1086/422649 15356813 10.1086/422649

[CR18] Iglói Z, Mughini-Gras L, Nic Lochlainn L, Barrasa A, Sane J, Mooij S et al (2018) Long-term sequelae of sporadic cryptosporidiosis: a follow-up study. Eur J Clin Microbiol Infect Dis 37:1377–1384. 10.1007/s10096-018-3268-9 29730717 10.1007/s10096-018-3268-9 PMC6015106

[CR19] Insulander M, Silverlås C, Lebbad M, Karlsson L, Mattsson JG, Svenungsson B (2013) Molecular epidemiology and clinical manifestations of human cryptosporidiosis in Sweden. Epidemiol Infect 141:1009–1020. 10.1017/S0950268812001665 22877562 10.1017/S0950268812001665 PMC9151846

[CR20] Khalil IA, Troeger C, Rao PC, Blacker BF, Brown A, Brewer TG, et al (2018) Morbidity, mortality, and long-term consequences associated with diarrhoea from *Cryptosporidium* infection in children younger than 5 years: a meta-analyses study. Lancet Glob Health 2018;6:e758-e68. 10.1016/S2214-109X(18)30283-3 10.1016/S2214-109X(18)30283-3 PMC6005120 29903377

[CR21] Kotloff KL, Nataro JP, Blackwelder WC, Nasrin D, Farag TH, Panchalingam S et al (2013) Burden and aetiology of diarrhoeal disease in infants and young children in developing countries (the Global Enteric Multicenter Study GEMS): a prospective case-control study. Lancet 382:209–222. 10.1016/S0140-6736(13)60844-2 23680352 10.1016/S0140-6736(13)60844-2

[CR22] Litleskare S, Rortveit G, Eide GE, Hanevik K, Langeland N, Wensaas KA (2018) Prevalence of irritable bowel syndrome and chronic fatigue 10 years after *Giardia* infection. Clin Gastroenterol Hepatol 16:1064–72.e4. 10.1016/j.cgh.2018.01.022 29378314 10.1016/j.cgh.2018.01.022

[CR23] Ludvigsson JF, Büsch K, Olén O, Askling J, Smedby KE, Ekbom A et al (2017) Prevalence of paediatric inflammatory bowel disease in Sweden: a nationwide population-based register study. BMC Gastroenterol 17:23. 10.1186/s12876-017-0578-9 28143594 10.1186/s12876-017-0578-9 PMC5282815

[CR24] Lupu VV, Ghiciuc CM, Stefanescu G, Mihai CM, Popp A, Sasaran MO et al (2024) Emerging role of the gut microbiome in post-infectious irritable bowel syndrome: a literature review. World J Gastroenterol 29:3241–3256. 10.3748/wjg.v29.i21.3241 10.3748/wjg.v29.i21.3241 PMC10292139 37377581

[CR25] MacKenzie WR, Schell WL, Blair KA, Addiss DG, Peterson DE, Hoxie NJ et al (1994) Massive outbreak of waterborne *Cryptosporidium* infection in Milwaukee, Wisconsin: recurrence of illness and risk of secondary transmission. Clin Infect Dis 21:57–62. 10.1093/clinids/21.1.57 10.1093/clinids/21.1.57 7578760

[CR26] Madadi S, Mahami-Oskouei M, Rafeey M, Spotin A, Aminisani N, Mahami-Oskouei L et al (2020) Comparative evaluation of *Cryptosporidium* infection in malnourished and well-nourished children: parasitic infections are affected by the interaction of nutritional status and socio-demographic characteristics. Comp Immunol Microbiol Infect Dis 68:101406. 10.1016/j.cimid.2019.101406 31881414 10.1016/j.cimid.2019.101406

[CR27] Mouratidou, N (2023) Childhood-onset inflammatory bowel disease – health care use, impact on growth and school achievements. Dissertation, Karolinska Institutet

[CR28] Ohlsson B (2022) Extraintestinal manifestations in irritable bowel syndrome: a systematic review. Therap Adv Gastroenterol 15:17562848221114558. 10.1177/17562848221114558 10.1177/17562848221114558 PMC9373179 35967918

[CR30] Pogreba-Brown K, Austhof E, Armstrong A, Schaefer K, Villa Zapata L, McClelland DJ et al (2020) Chronic gastrointestinal and joint-related sequelae associated with common foodborne illnesses: a scoping review. Foodborne Pathog Dis 17:67–86. 10.1089/fpd.2019.2692 31589475 10.1089/fpd.2019.2692 PMC9246095

[CR31] Rogler G, Zeitz J, Biedermann L (2016) The search for causative environmental factors in inflammatory bowel disease. Dig Dis 34(Suppl 1):48–55. 10.1159/000447283 27548430 10.1159/000447283

[CR32] Sjöström M, Arvidsson M, Söderström L, Lilja M, Lindh J, Widerström M (2022) Outbreak of *Cryptosporidium hominis* in northern Sweden: persisting symptoms in a 5-year follow-up. Parasitol Res 121:2043–2139. 10.1007/s00436-022-07524-5 35451705 10.1007/s00436-022-07524-5 PMC9192462

[CR33] Skovgaards DM, Hartmeyer GN, Skov MN, Hoegh SV, Kemp M (2018) *Cryptosporidium* species are frequently present but rarely detected in clinical samples from children with diarrhea in a developed country. Pediatr Infect Dis J 37:e138–e140. 10.1097/INF.0000000000001794 28938260 10.1097/INF.0000000000001794

[CR34] Vandenberg O, Robberecht F, Dauby N, Moens C, Talabani H, Dupont E et al (2012) Management of a *Cryptosporidium hominis* outbreak in a day-care center. Pediatr Infect Dis J 31:10–15. 10.1097/INF.0b013e318235ab64 22094626 10.1097/INF.0b013e318235ab64

[CR35] Widerström M, Schönning C, Lilja M, Lebbad M, Ljung T, Allestam G et al (2014) Large outbreak of *Cryptosporidium hominis* infection transmitted through the public water supply, Sweden. Emerg Infect Dis 20:581–589. 10.3201/eid2004.121415 24655474 10.3201/eid2004.121415 PMC3966397

[CR36] World Health Organization (2017) Diarrhoeal disease. https://www.who.int/news-room/fact-sheets/detail/diarrhoeal-disease. Accessed 10 Feb 2023

[CR37] Young I, Smith BA, Fazil A (2015) A systematic review and meta-analysis of the effects of extreme weather events and other weather-related variables on *Cryptosporidium* and *Giardia* in fresh surface waters. J Water Health 13:1–17. 10.2166/wh.2014.079 25719461 10.2166/wh.2014.079

