# Supplementary material for: Long-term symptoms in children after a Cryptosporidium hominis outbreak in Sweden: a 10-year follow-up
Source: Parasitol Res. 2025 Jan 25;124(1):13. doi: 10.1007/s00436-025-08455-7 (PMC11762772; doi:10.1007/s00436-025-08455-7)
Supplement: Supplementary file 1 — Supplementary file1 (PDF 162 KB) [file 436_2025_8455_MOESM1_ESM.pdf]

Survey: Follow-up after the Cryptosporidium outbreak in winter 2010/2011 (Children)
(Tick the boxes using a ballpoint pen)

Child's age: ___ years

The child is a: [ ] Boy [ ] Girl

1a. During the past three months, has your child experienced any of the following symptoms?

	Yes	No
- Diarrhoea with 3 or more loose stools per day	[ ]	[ ]
- Watery diarrhoea	[ ]	[ ]
- Bloody diarrhoea	[ ]	[ ]
- Abdominal pain/cramps	[ ]	[ ]
- Vomiting	[ ]	[ ]
- Nausea	[ ]	[ ]
- Fever, more than 38°C	[ ]	[ ]
- Headache	[ ]	[ ]
- Joint discomfort	[ ]	[ ]
- Eye pain	[ ]	[ ]
- Fatigue	[ ]	[ ]

(If you have answered "No" to all the above, please proceed to question 2a)

1b. For how many days during the past three months did your child experience any of the symptoms in question 1a?

Number of days with symptoms: ___

1c. Do you believe any of the symptoms in question 1a started after the Cryptosporidium outbreak?

[ ] Yes, some of the symptoms started then
[ ] No, the symptoms did not start then

2a. During the past three months, has your child had any of the following symptoms?

	Yes	No
- Constipation		
- Altering bowel habits (constipation/diarrhoea)	[ ]	[ ]
- Bloating	[ ]	[ ]
- Acid indigestion	[ ]	[ ]
- Loss of appetite	[ ]	[ ]
- Weight loss	[ ]	[ ]

2b. If your child experienced any symptoms from question 2a, how many days have these symptoms occurred in the past 3 months?

Number of days: __

3a. During the past three months, has your child experienced any of the following symptoms?

	Yes	No
- Stiff joints	[ ]	[ ]
- Joint pain	[ ]	[ ]
- Swollen joints	[ ]	[ ]

3b. If your child experienced any symptoms from question 3a, how many days have these symptoms occurred in the past 3 months?

Number of days: ___

4. During the past three months, has your child been absent from daycare/school due to symptoms mentioned in question 2a or 3a?

[ ] Yes
[ ] No

If yes, for how many days in total? ___ days

5. During the past three months, has your child sought medical care for symptoms mentioned in question 2a or 3a?

Check one or more options:

[ ] No
[ ] Yes, primary care center
[ ] Yes, hospital

6. Does your child have any known food intolerances or sensitivities?

(Intolerances that have caused stomach or bowel problems, e.g., loose stools, stomach pain, bloating)

	Yes	No
- Cow's milk allergy	[ ]	[ ]
- Lactose intolerance	[ ]	[ ]
- Gluten intolerance	[ ]	[ ]
- Other known food intolerance	[ ]	[ ]

7. Does your child have any of the following illnesses?

	Yes	No
- Diabetes	[ ]	[ ]
- Inflammatory bowel disease	[ ]	[ ]
(Ulcerative colitis or Crohn's disease)		
- Irritable bowel syndrome (IBS)	[ ]	[ ]
- Rheumatic joint disease	[ ]	[ ]

8. Is your child currently being treated with any of the following medications?

	Yes	No
- Medication for ulcers/acid reflux	[ ]	[ ]
(e.g., Omeprazole, Losec, Nexium)		
- Cortisone tablets	[ ]	[ ]

9. Do you <u>think</u> your child is currently experiencing symptoms caused by the Cryptosporidium infection from 2010-2011?

[ ] Yes
[ ] No

If yes, please provide comments and information on the back of this paper.

10. Are you concerned about your child's health?

Circle the number that best represents your level of concern, where 0 means "Not concerned at all" and 10 means "Very concerned."

Not concerned at all 0 1 2 3 4 5 6 7 8 9 10 Very concerned

11. Did your child have a Cryptosporidium infection in 2010/2011?

[ ] Yes
[ ] No
[ ] Don't know

Thank you for taking the time to answer the questions! We would appreciate it if you could return your response as soon as possible in the enclosed reply envelope. Feel free to add any comments or information on the back or on a separate sheet of paper.

(Translation for publication purposes only, layout differs from the Swedish questionnaire.)
