# Supplementary figures and images for: Long-term symptoms in children after a Cryptosporidium hominis outbreak in Sweden: a 10-year follow-up
Source: Parasitol Res. 2025 Jan 25;124(1):13. doi: 10.1007/s00436-025-08455-7 (PMC11762772; doi:10.1007/s00436-025-08455-7)

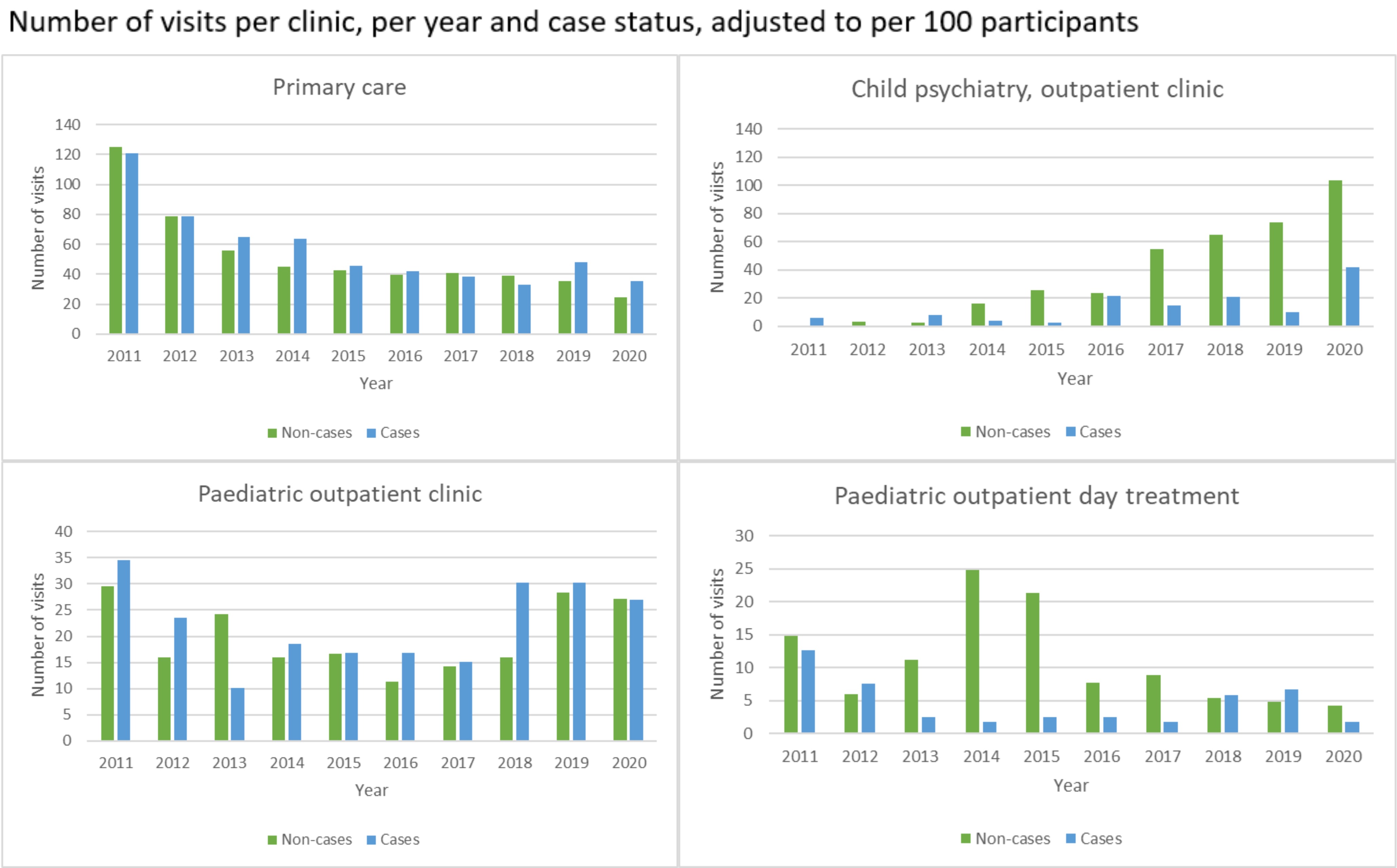

Supplement: Supplementary file 2 — Supplementary file2 (JPG 482 KB) [file 436_2025_8455_MOESM2_ESM.jpg]
